# Meeting the Challenge of Diabetes in China

**DOI:** 10.15171/ijhpm.2019.80

**Published:** 2019-10-06

**Authors:** Zhen Luo, Guilhem Fabre, Victor G. Rodwin

**Affiliations:** ^1^Robert F. Wagner School of Public Service, New York University (NYU), New York City, NY, USA.; ^2^IRIEC EA 740, Université Paul Valéry, Montpellier3, Montpellier, France.; ^3^World Cities Project, Wagner School of Public Service, New York University (NYU), New York City, NY, USA.

**Keywords:** China, Diabetes, Health Policy, Artificial Intelligence, Big Data

## Abstract

China’s estimated 114 million people with diabetes pose a massive challenge for China’s health policy-makers who have significantly extended health insurance coverage over the past decade. What China is doing now, what it has achieved, and what remains to be done should be of interest to health policy-makers, worldwide. We identify the challenges posed by China’s two principal strategies to tackle diabetes: (1) A short-term pilot strategy of health promotion, detection and control of chronic diseases in 265 national demonstration areas (NDAs); and (2) A long-term strategy to extend health promotion and strengthen primary care capacity and health system integration throughout China. Finally, we consider how Chinese innovations in artificial intelligence (AI) and Big Data may contribute to improving diagnosis, controlling complications and increasing access to care. Health system integration in China will require overcoming the fragmentation of a system that still places excessive reliance on local government financing. Moreover, what remains to be done resembles deeper challenges faced by healthcare systems worldwide: the need to upgrade primary care and reduce inequalities in access to health services.

## Introduction

China accounts for the largest number of people with diabetes (114 million),^1^ mainly type 2, within a single nation. This poses a massive challenge for China’s policy-makers who have significantly extended health insurance coverage over the past decade. Medical management of diabetes, alone, before complications, is already estimated to account for 8.5% of national health expenditure in China.^1-3^ As Professor Linong Ji, Vice President of the International Diabetes Federation averred, if diabetes is not controlled in China, “the wealth that we’ve accumulated over the past 30 years will be wasted in the drains of our dialysis machines.”^4^

What China is now doing, what it has achieved, and what remains to be done, can be instructive for health policy-makers, worldwide. Based on journal articles in Chinese, government sources and peer-reviewed research articles, in English, we review prevalence estimates, variations by geographic areas, and assess China’s accomplishments and the two principal strategies it has implemented to date: (1) A short-term pilot strategy of health promotion, detection and control of chronic diseases in 265 national demonstration areas (NDAs); and (2) A long-term strategy to extend health promotion and strengthen primary care capacity and health system integration among local communities and health providers, including hospitals, throughout China. We also consider how Chinese innovations in artificial intelligence (AI) and Big Data may contribute to health promotion, detection and management of diabetes in the future.

## Diabetes Prevalence: National Average and Variations

Prevalence estimates of disease are highly dependent on the assumptions, definitions and survey methods used, all of which require judgment calls and interpretation. Thus, it is not surprising that five recent surveys, in China, arrive at different estimates of diabetes prevalence (Table 1). Although these surveys were conducted at different times, the differences may also reflect different methods (including selection of areas and samples, sample size, blood glucose testing indicators, population age structure) rather than disease prevalence. For example, the 1994 survey^5^ was not based on a nationally representative sample and the two most recent surveys (2010 and 2013)^6,7^ apply 2010 criteria from the American Diabetes Association’s (ADA’s) definition, while the 2007-2008 and 1994 surveys applied more restrictive criteria from the World Health Organization (WHO).^5,8^

**Table 1:** National Surveys of Diabetes in China

**Year of the Survey**	**Prevalence Estimate (%)**	**Detection Rate (Patients Aware of Their Condition) (%)**	**Prediabetes Prevalence Estimate (%)**	**Source**
1994^a^	2.51	29.6	3.23	Pan et al, 1997^5^
2000-2001^b^	5.49	-	7.33	Hu et al, 2009^9^
2007-2008^c^	9.7	39.3	15.5	Yang et al, 2010^8^
2010^d^	11.6	30.1	50.1	Xu et al, 2013^6^
2013^e^	10.9	36.5	35.7	Wang et al, 2017^7^

^a^ A survey based on a sample of 250 000 adults aged 25 years or older, in 19 areas, including cities and rural areas of the north, south, east, west, and middle part of China. Diagnostic criteria from WHO, 1985. ^b^ A survey based on a population of 19 012 adults, aged 35-74 and randomly selected from 20 primary sampling units (street districts in urban areas, townships in rural areas) and invited to participate. Diagnostic criteria from ADA. ^c^ A survey based on a nationally representative sample of 46 239 adults, 20 years of age or older, from 14 provinces and municipalities participated in the study. Diagnostic criteria from WHO, 1999. ^d^ A cross-sectional survey in a nationally representative sample of 98 658 adults. Diagnostic criteria from the ADA, 2010. ^e^ A nationally representative cross-sectional survey of 170 287 adults; minority ethnic groups in China with at least 1000 participants (Tibetan, Zhuang, Manchu, Uyghur, and Muslim) were compared with Han participants. Diagnostic criteria from the ADA, 2010.

Even after taking into account methodological differences among these surveys, the four-fold growth in diabetes prevalence since 1994 is striking (Table 1).^5,7^ The socio-economic and cultural determinants of this growth are most often attributed to the transformation of diet,^10^ genetic factors,^11^ the effects of the Great Famine (1958-1962)^12,13^ and its intergenerational effects.^13^ In Chinese megacities, children are typically raised by their grandparents, most of whom experienced the famine. This experience has likely contributed to their high-calorie intake and low physical activity, and in this way exacerbated the intergenerational transmission of risk factors for diabetes.

Another striking finding from the surveys is the low share of people with diabetes who are aware of their illness (the detection rate), a significant indicator since diabetes patients who do not control their blood sugar levels are likely to end up with cardiovascular disease or kidney failure. The most recent survey^7^ indicates a 36.5% detection rate in 2013, which is much lower than in France (67.24%),^14^ the United States (75.65%),^15^ the United Kingdom (83.55%),^16^ Australia (71%),^17^ and India (47.98%).^18^

### Geographic Variation

There are enormous variations in diabetes prevalence among urban and rural areas and wealthy and poorer regions. In 2016, urban versus rural residents’ annual income (33 616 yuan) was almost 3 times that of their rural counterparts (12 363 yuan).^19^ Based on the 2010 survey, prevalence of diabetes ranges from 14.3% in urban versus 10.3% in rural areas.^6^ Higher-income urban counties have a higher average prevalence rate (13.1%), than rural low-income counties (8.7%)^20^; or according to a 2013 survey, 12.6% in urban versus 9.5% in rural areas.^7^ Based on a 2010 survey, adults with diabetes living in rural areas are less likely to be aware of their illness (38.7%) than their counterparts in urban areas (24.6%)^6^; according to another 2010 survey, the detection rate ranges from 40.8% in higher-income urban counties to 20.5% in lower-income rural counties^20^; according to the 2013 survey, 43.1% in urban and 29.1% in rural areas.^7^ In general, people residing in wealthier urban areas are more likely to have diabetes. Among the poorest populations in these areas, however, due to a lower level of health awareness, they face lower odds of having their condition detected and managed.^20^

### Migrant Workers and Urban-Rural Variation

China has experienced the world’s most rapid urbanization over the past two decades. Attracted by higher wages, millions of rural residents have migrated to cities. Those who have left their hometown for more than 6 months and have not been able to register as official residents in cities are considered migrant workers. Although their number has declined from a peak of 253 million (in 2014), to 245 million migrants (in 2016),^21^ they still pose a public health challenge since most municipal governments regard them as outsiders and their health insurance benefits (from home provinces) are typically insufficient to assure them of access to healthcare in cities.

Although migrant workers face fewer risk factors for diabetes, since most healthy migrants participate in heavy labor,^22^ once they become diabetic, or even prediabetic, they are more likely to forgo medical care due to financial barriers than official urban residents.^23^ Since diabetes is associated with increased mortality from a range of complications, their health status is likely to deteriorate faster than that of their official urban counterparts.^22^ Given the low diabetes detection rates (Table 1) and the low treatment rates for those diagnosed with diabetes, there is evidence of excess mortality of diabetes patients, which points to inadequate health promotion and clinical management of diabetes.^24,25^

## National Strategies: Achievements and Problems

In the Healthy China 2030 Plan, approved by the State Council and the Party’s Central Committee, diabetes, along with cancer, hypertension, and cardiovascular diseases, are listed as the four major non-communicable diseases (NCDs) for which the goal is to “control the prevalence and reduce the probability of early death.”^26^ Also, in 2017, the central government launched a 2017-2025 plan to control NCDs, which includes increasing the regular monitoring and self-management rate for diabetes patients from 50% (which is probably an overestimate) to 70% by 2025.^27^ To achieve population behavior change in any society is a difficult task; it is all the more so in large and diverse countries like China. Two national strategies, in particular, deserve special attention.

### National Demonstration Areas: A Short-term Strategy

Since 2010, the Chinese Ministry of Health has established a network of 265 NDAs to develop pilot projects for health promotion, detection and control of chronic diseases. To qualify for an NDA, county governments applied for central government subsidies and agreed to follow strict protocols. As of 2015, 9.29% of China’s counties established NDAs under the responsibility of China’s Center for Disease Control (CDC). At the county level, various local government departments (propaganda, women’s federation, education, health) are involved and in most cases the top county official is responsible for overall coordination. Primary care providers promote education and self-management of health conditions through appropriate dietary changes, control of tobacco use, and physical activity.^28^

A 2018 evaluation on the implementation of NDAs compared the 265 NDAs to one another along multiple criteria to assess their effectiveness in managing patients with diabetes and other NCDs.^29^ Aside from disparities across the NDAs, it found that health promotion activities and monitoring general population health were generally more successful than managing the conditions of those suffering from NCDs. With regard to diabetes, this conclusion most probably applies to all of China as it is consistent with the findings of Bragg and colleagues on the high proportion of deaths attributed to diabetic ketoacidosis or coma and chronic kidney disease mainly related to diabetes.^24^ These deaths are usually related to poor clinical management.

Another 2018 evaluation of the NDAs, specifically on the management of patients with diabetes, by Jin and colleagues, suggests that health promotion efforts resulting in lifestyle changes and self-management can nevertheless improve process measures of diabetes control.^30^ This study, conducted in 2016, on 3213 randomly selected adult residents (35 years and over) in NDAs, found that, among patients diagnosed with diabetes, 69.92% were assigned to a general practitioner (GP) and 53.66% reported receiving standardized management for their condition – at least one blood glucose test and four follow-up visits, per year, by a GP. It found that patients who are assigned to a GP, have a more positive attitude, and that patients without hypertension, are more likely to have their diabetes under better standardized control, ie, with patients receiving at least one blood glucose test and four follow-up visits, per year, by a GP. The study also found that patients in eastern provinces are under better standardized control, which could be the result of earlier establishment of NDAs; or alternatively, could be attributed to better health systems in these provinces.^30^ While the results of NDAs are encouraging, they also highlight the need for greater centralization of funding for public health interventions. The 2018 plan indicates that health spending has depended largely on local governments, which explains the wide disparities in access to health services among counties.^31^

Jin and colleagues conclude that “NDAs have achieved the goals of the 2012-2015 National NCD Control Plan, which requires a 40% standardized management rate.”^30^ They also find that 79.54% of patients in their study were aware of their diabetes diagnosis and 83.2% were in taking prescription drugs regularly to control their blood glucose levels. Both of these figures are high for China which suggests that NDAs have produced significant effects. It is difficult, however, to interpret these figures for several reasons. First, there were no control groups in this study – neither cross-sectional, nor before and after the intervention. They relied on comparisons with other national surveys that may not be comparable to theirs. Second, their survey question on self-reported high blood sugar levels is ambiguous. It is not sufficient to confirm the diagnosis and may include prediabetes patients. In fact, Jin and colleagues suggest that further surveys be conducted to see if standardized management has affected blood sugar control. Thus, we believe that this evaluation must be read with caution. Nonetheless, the NDAs represent important pilots for learning about disparities in prevalence, awareness and treatment of diabetes. Moreover, they are important for testing health promotion and monitoring tools, self-management and standardized management approaches for controlling diabetes.

### Health System Integration: A Long-term Strategy

Beyond the importance of pilot projects, the long-term strategy for confronting the diabetes challenge in China is to extend efforts at health promotion, detection and control of diabetes while simultaneously integrating academic medical centers (AMCs) and other hospitals with effective primary care. AMCs are overcrowded with patients who have simple conditions, while the primary care system is under-equipped with basic medical infrastructure and an under-qualified workforce. Since 2015, the State Council encouraged health system integration by promoting what the Chinese call a “hierarchical medical system,”^32^ a policy that attempts to attract more patients to primary health centers before going to AMCs.

The aspiration to achieve health system integration, in China, is as daunting as in any other health system. Moreover, China faces two significant obstacles. First, most primary care institutions are not qualified enough to earn patients’ trust. In 2017, 82% of physicians and assistant physicians in rural township health centers and 55% in urban community health centers have no bachelor’s degree, in contrast to 70% of their counterparts in hospitals^[1]^.^2^ In the absence of uniform professional standards, it is rational for patients to crowd into the best hospitals. Second, under the current system, hospitals have more abundant supplies of prescription drugs, partly because selling them is still an important part of hospital revenue. Although overcrowded, AMCs have little incentive to divert patients to primary care institutions.

In confronting these obstacles, municipal governments in big cities are attempting to integrate AMCs and community clinics into so-called medical partnerships. There are several models for achieving such health system integration. Some partnerships share only the same referral system; others have actually achieved deeper cooperation such that different institutions share the same workforce and operate under the same leadership. In general, cooperation is stronger in big cities than in rural areas. Shenzhen’s *Luohu model* is an example of high-level of cooperation where primary care institutions and AMCs form a “hospital group” under the leadership of a common board of directors. Medical partnerships in big cities often resemble the *Luohu model* , though the level of cooperation varies.^33^

## The Future

Although the implementation of NDAs and efforts to achieve health system integration are both well-conceived and necessary steps to confront the challenge of diabetes and more generally achieve China’s goals for healthcare reform, these interventions have so far been inadequate. Two deeper long-term challenges remain before significant progress can be made in stemming the diabetes crisis: upgrade the primary care workforce; and reduce inequalities in access to healthcare.

### The Primary Care Workforce

Long-term management of diabetes requires a competent primary care workforce backed up by available resources. However, these requirements are not yet met in China, neither for people with diabetes nor for the general population. Five percent of urban community health centers and 10% of township (rural) health centers are unable to provide basic services such as routine blood tests, urinalyses and electrocardiography. For village clinics, 96% are unable to provide routine blood tests, 90% are unable to provide urinalyses, and 31% are unable to provide blood glucose tests. Only 46% of patients with diabetes who usually seek care from primary healthcare institutions are diagnosed, and only 3% of them have their blood glucose controlled. Among all patients treated by primary care institutions, 84% are not satisfied and 39% of patients who bypassed primary care institutions noted their distrust as the reason for their choice.^34^

In 2016, the ratio of certified GPs per 10 000 population was 1.6, far below high-income countries’ average (Table 2). As China has sought to improve health system integration through the rapid expansion of the primary care workforce, the number of certified GPs has increased by 34% but the physician workforce in hospitals increased faster (by 57%) than urban (15%) and rural (37%) primary care institutions.^2^

**Table 2:** Primary Care Workforce: China and Other Countries, 2016

	**GPs (Per 1000 Population)**	**Nurses (Per 1000 Population)**
China	0.16	2.32
Korea	0.13	6.80
Japan	-	11.34
US	0.31	11.61
Australia	1.17	11.57
UK	0.76	7.88
Canada	1.27	9.91
France	0.90	10.21
Germany	0.70	12.84

Abbreviation: GPs, general practitioner. Source: National Commission of Health and Family Planning Statistical Yearbook^3^ and OECD data.^35^

Unfortunately, GPs in China remind the population of barefoot doctors in the Mao era, during which professional standards of medicine were severely compromised to achieve universal coverage of basic necessities. Due to the transformation of China’s healthcare system over the past decades, the term “doctor” refers to a wide range of professionals, from village doctors with months of training to graduates of the best medical schools.^36^ Although China is trying to reestablish professional standards through reforms in medical education, it will take time before the profession of GP becomes an attractive one for medical students and wins the trust of patients.

### Inequalities in Access to Healthcare

Despite the proclaimed commitment to universal health coverage, as we noted earlier, the vast majority of China’s migrants are covered by their home provinces, which provide them much less generous insurance coverage than their resident cities. Though efforts have been made to increase reimbursement in their resident cities, due to administrative barriers, it is still difficult for migrant workers to pay out-of-pocket for healthcare and then wait to be reimbursed a small share of their outlays.^37^ This situation is most severe in large cities with the highest prevalence of migrants and diabetes. For example, almost a third of Beijing’s 21.71 million residents (8.08 million) do not have an urban residence card (hukou).^38^ The same situation prevails in Shanghai where 9.7 million residents, of its 24.2 million inhabitants, have no urban residence card.^39^

In these wealthy cities, since migrants are, on average, living in more precarious conditions, less educated, and less attentive to potential health problems, they tend to have higher than average rates of overweight and obesity and alcohol consumption. Their risk of diabetes is therefore higher than that of their official urban counterparts.^22^ Despite these well-recognized problems, the Beijing and Shanghai 2030 Health Plans do not include specific measures for diabetes control among migrants.^40,41^

## Artificial Intelligence and Big Data

Beyond the importance of taking action on health promotion strategies to improve diet, encourage exercise and increase greater awareness of risk factors for diabetes, China’s health policy-makers have not overlooked significant advances in AI and Big Data.^42^ In some respects, the future is already here with respect to automated retinal screening (detection of diabetic retinopathy), clinical decision support (detection and monitoring of diabetes and comorbidities), predictive population risk stratification (identification of diabetes subpopulations at higher risk of complications) and patient self-management tools (eg, activity and dietary tracking devices and AI-improved glucose sensors). As these tools are improved, they will soon enable health professionals in China to identify some prediabetic patients and to diagnose complications related to diabetes such as retinopathy and renal diseases.^43^ On-going research on specific algorithms may also eventually allow more personalized targeting of diabetes treatment.^44^

Today, diabetes patients are generally more “connected” than patients with other chronic conditions. Activity trackers and smartphone applications have the potential to prevent type 2 diabetes by promoting the adoption of behaviors that prevent the onset of the disease, such as physical activity and dietary changes. Also, use of connected sensors or mobile applications already allow better daily monitoring of patients with type 2 diabetes.^45^ In addition to introducing innovations such as intelligent sensors for measuring and treating blood glucose levels, Ali Health, the medical branch of the giant Alibaba launched an AI physician system, Dia Doc, in May 2018. This system, dedicated to diabetes, brings together experienced specialists and practitioners, and guides GPs less familiar with the disease in performing diagnosis and treatment.^46^

China’s digital transformation and its massive amounts of accumulated data provide an opportunity for the health system to leverage AI capabilities in improving diabetes care.^47^ Diet and exercise, two fundamental risk factors for most NCDs, are under the surveillance of Chinese tech-giants. E’leme (Are You Hungry?), a takeaway app recently acquired by the top tech-giant, Alibaba, has 260 million users, and Ali has already been collecting data on its users’ diets for years.^48^ Ali’s competitor Tencent, whose most successful app, WeChat has 1.08 billion users who use it at least once a month,^49^ has been collecting data on the average number of steps, per day, walked by the Chinese.^50^

Drawing on their subsidiary payment systems, Alipay and WeChat Pay, both Ali and Tencent are capable of analyzing nearly all aspects of the population’s economic activities. Their ambition clearly extends beyond expanding membership, for health improvement strategies present a most desirable new frontier, especially since the government is encouraging the use of Big Data in the Healthy China 2030 development plan.^26^ Health was one of the top priorities in the government’s plan to promote the use of Big Data and encourage cooperation among technology enterprises as a way to confront diabetes and other NCDs.^51^ Premier Li Keqiang, himself, has flagged Big Data as an area from which the public health sector should learn.^52^

## Concluding Observations

Faced with the world’s largest population of people with diabetes that continues to grow with China’s rapid urbanization, China has significantly improved the detection and management of diabetes, with its pilot projects in NDAs and its attempts to improve health system integration. These initiatives demonstrate a willingness to confront the rise of diabetes with strategies based on health promotion, disease detection and management of the multiple conditions and complications associated with the disease. AI and Big Data are likely to improve diagnosis and treatment. However, there is no nation in which the challenge of diabetes is likely to be solved by a technological fix. AI and Big Data must be financed and embedded in an institutional health system. Based on the literature and data we have reviewed and analyzed in this paper, significant challenges remain.

The extension to the rest of China of lessons learned from short-term strategies implemented in NDAs will require overcoming the fragmentation of a health system that still places excessive reliance on local government financing. Ongoing reform of the health system will require greater efforts to train and pay primary care providers to relieve a system that is too focused on large hospitals, and to achieve broader health system integration. Above all, longer-term success in meeting the challenge of diabetes will have to focus on upgrading the primary care workforce and reducing inequalities in access to healthcare.

## Acknowledgements

We thank Manuel Raices Perez-Castañeda for his invitation to present a preliminary version of this paper to the International Diabetes Congress (IDC) in Varadero, Cuba in December, 2018. We are also grateful to Dr. Zhicheng Wang, School of Public Health, Peking University, Beijing, China, who provided useful information as we began our research. Finally, we thank our anonymous reviewers for most helpful suggestions and Wagner/NYU, New York City, NY, USA and University Paul Valéry-Montpellier 3 (IRIEC), Montpellier, France for travel support to attend the IDC.

## Ethical issues

Not applicable.

## Competing interests

Authors declare that they have no competing interests.

## Authors’ contributions

ZL and GF conducted the main documentary research for this paper within the overall framework developed by VGR. All three authors participated in drafting the paper and revising it for final submission.

## Authors’ affiliations

^1^Robert F. Wagner School of Public Service, New York University (NYU), New York City, NY, USA. ^2^IRIEC EA 740, Université Paul Valéry, Montpellier3, Montpellier, France. ^3^World Cities Project, Wagner School of Public Service, New York University (NYU), New York City, NY, USA.

## Endnote


^[1]^In China, healthcare institutions are categorized as hospitals, urban community health centers and rural township health centers. Urban community and rural township health centers are considered as primary care institutions, but they also provide inpatient care. The yearbook does not provide strict and clear definitions but it is generally believed that hospitals provide comprehensive care while urban and rural health centers are smaller than hospitals and provide mainly primary care.
